# An Autopsy Case of Neurofibromatosis Type 1 Mutation Detected in a Duodenal Lesion of Malignant Melanoma: A Case Report

**DOI:** 10.7759/cureus.90933

**Published:** 2025-08-25

**Authors:** Mai Seki, Emi Saitou, Shintaro Saito, Takeshi Araki, Yoichiro Shinohara, Kazuyoshi Fujihira, Tetsunari Oyama, Hideaki Yokoo

**Affiliations:** 1 Diagnostic Pathology, Gunma University Graduate School of Medicine, Maebashi, JPN; 2 Dermatology, Gunma University Graduate School of Medicine, Maebashi, JPN; 3 Ophthalmology, Gunma University Graduate School of Medicine, Maebashi, JPN; 4 Psychiatry and Neuroscience, Gunma University Graduate School of Medicine, Maebashi, JPN; 5 Human Pathology, Gunma University Graduate School of Medicine, Maebashi, JPN

**Keywords:** duodenum, malignant melanoma, nf1, sox-10, von recklinghausen disease

## Abstract

We report an autopsy case of duodenal malignant melanoma (MM) in a patient with neurofibromatosis type 1 (NF1). The patient had an anamnesis of choroidal MM, and the duodenal lesion was initially suspected to be a metastasis from the choroidal tumor. At autopsy, the duodenum was occupied by a tumor composed of large atypical cells on histopathological examination. Immunohistochemically, the nuclei of large atypical cells were positive for Melan A, HMB-45, and SOX-10, confirming the diagnosis of MM. Next-generation sequencing (NGS) of the duodenal tumor sample identified an NF1 mutation, with no other gene mutations clearly associated with malignant transformation.

## Introduction

Recently, neurofibromatosis type 1 (NF1) mutations have been reported in patients with malignant melanoma (MM). Meyer et al. [1] reported that MM occurred in 43.6% of patients with NF1 mutations, although the risk of melanoma in this population remains incompletely understood. We report a rare autopsy case of duodenal melanoma in a patient with neurofibromatosis and a history of choroidal MM. Both lesions were diagnosed histologically as MM, a rare condition, and were suspected of being duodenal metastases from a choroidal melanoma.

This is an autopsy case of a deceased patient, and consent was obtained from the patient's family on May 23, 2024.

## Case presentation

**Figure 1:**
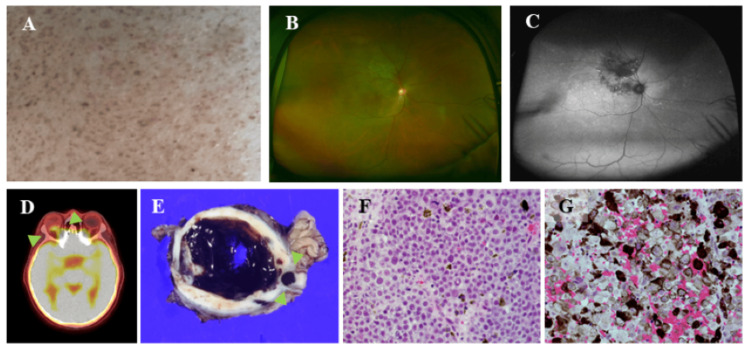
Café-au-lait spots on the skin (A) Café-au-lait spots were observed from the shoulders to the back. A color fundus photograph shows (B) a mass lesion with serous retinal detachment in the superior temporal region of the optic nerve head, along with (C) fundus autofluorescence. (D) Fluorodeoxyglucose uptake, indicated by a maximum standardized uptake value (SUVmax) of 4.5 in the right choroid, showed accumulation of uncertain malignant or inflammatory nature. (E) Macroscopically, a small black solid tumor was found adjoining the optic nerve. The tumor consisted of two areas: (F) one with tumor cells poor in melanin pigment and (G) another with tumor cells containing abundant melanin pigment within the cytoplasm

A 32-year-old male with visual impairment presented to our hospital for medical evaluation and treatment. His medical history included leukemia and adjustment disorder. Café-au-lait spots on the skin led to a clinical diagnosis of NF1 (Figure 1A).

**Figure 2:**
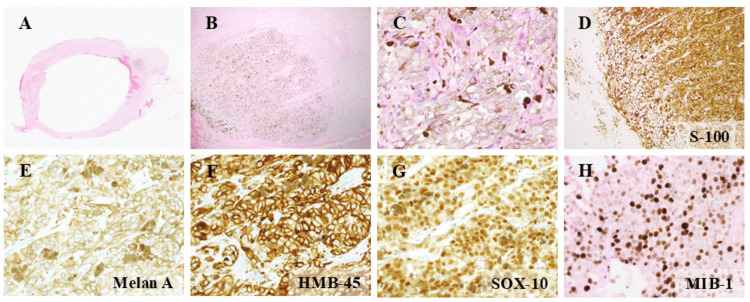
Histological analysis (A, B) The tumor was located near the right optic nerve. (C) Pleomorphic atypical tumor cells were observed, with mitotic activity and melanin pigmentation. (D) Both tumor cells and optic nerve cells were positive for S-100. (E–H) Tumor cells also stained positive for Melan A, HMB-45, and SOX-10 and showed an MIB-1 index of 47%

A mass lesion with serous retinal detachment in the superior temporal region was observed in the superior temporal region of the optic nerve head, along with fundus autofluorescence (Figures 1B, 1C). Fluorodeoxyglucose uptake, indicated by a maximum standardized uptake value (SUVmax) of 4.5 in the right choroid, showed accumulation of suspected malignant or uncertain inflammatory nature (Figure 1D). A biopsy confirmed the diagnosis of choroidal MM. The tumor was completely resected, and surgical margins were negative by pathological test. Macroscopically, a small solid tumor was located near the right optic nerve (Figure 1E). Pathological analysis of the specimen is shown in Figures 1F, 1G, and Figure 2.

The tumor consisted of large atypical cells, with 0-1 mitotic figures observed per high-power field (x400). Immunohistochemically, the tumor was positive for S-100, Melan A, HMB-45, and SOX-10. MIB-1 positivity was observed in about 47% of the tumor cells (Figure 2).

**Figure 3:**
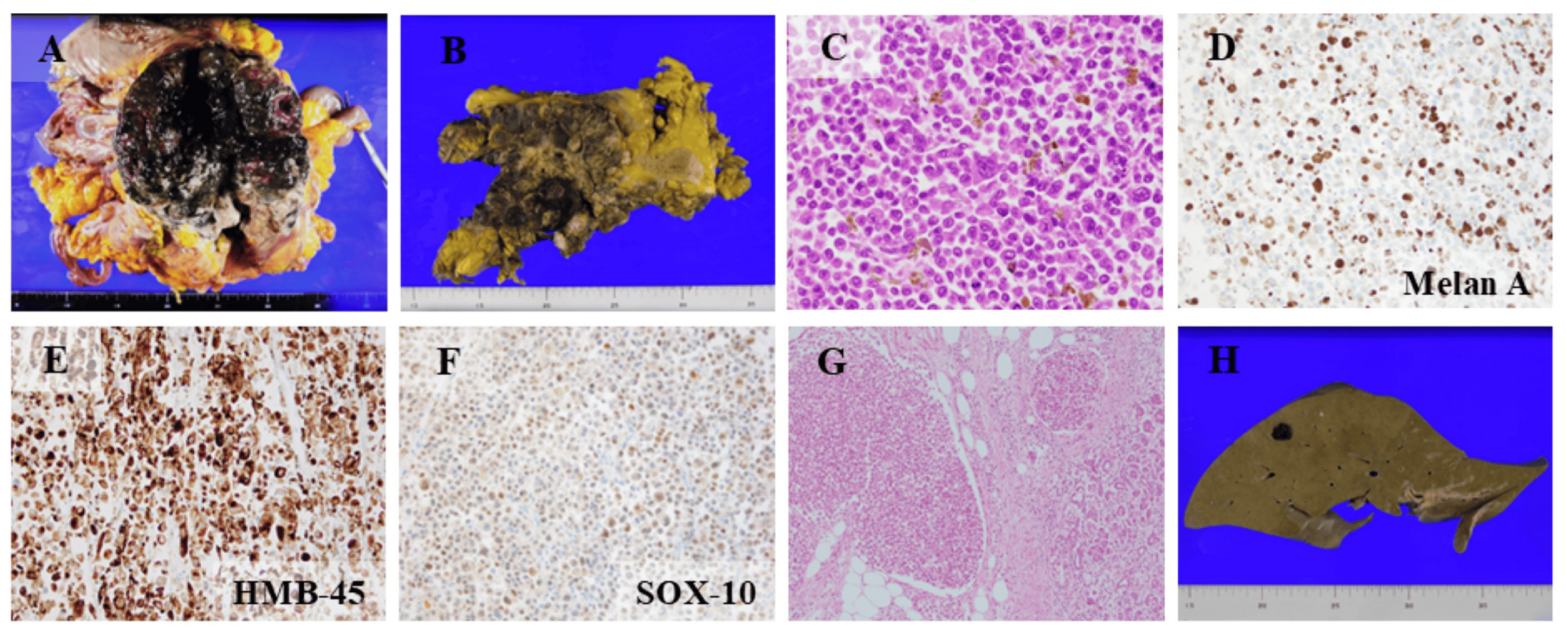
Autopsy - pathological and immunohistochemical findings (A) Duodenal tumor at autopsy. (B) The tumor had spread widely and invaded the pancreas. (C) A black metastatic lesion was observed in the liver after formalin fixation. (C) The tumor was composed of atypical cells, and melanin was present in some tumor cells. (D–F) Immunostaining of the tumor cells was positive for Melan A (D), HMB-45 (E), and SOX10 (F). (G) The tumor had spread to the pancreatic tissue. (H) Macroscopically, a black tumor nodule was observed in the liver

Eight months later, multiple liver metastases were detected during follow-up. PET of the liver metastases showed a high SUVmax of 13.71. To guide chemotherapy selection, cancer panel testing was performed; however, no BRAF mutation was identified. Chemotherapy with nivolumab and ipilimumab was administered; however, duodenal and abdominal lymph node metastases appeared one year later. The SUVmax of the duodenal lesions was also high, at 11.8. Furthermore, despite treatment with dacarbazine, the lesions progressed, and hepatic dysfunction developed. The patient died four years after initiating chemotherapy. An autopsy was performed to investigate the cause of repeated hematemesis and melena. A full-body autopsy, including the brain, was conducted. The tumor occupied the duodenum and had spread around the pancreas (Figures 3A, 3B).

The tumor was composed of atypical cells, and meganucleated and multinucleated tumor cells were observed (Figure 3C). The morphology of the atypical cells was similar, though the number of mitotic figures was increased in the duodenum compared with the choroidal lesion. Melanin was also present in some of the tumor cells, and melanophages had infiltrated the surrounding area. Increased mitotic activity and atypical mitotic figures were also observed. Five mitotic figures were observed per high-power field. Immunostaining of the tumor cells was positive for S-100, Melan A, HMB-45, and SOX10, consistent with MM (Figures 3D-3F). The tumor had spread widely around the duodenum, infiltrating the pancreatic tissue and stomach (Figure 3G). Multiple metastases were also observed in the lymph nodes around the duodenum and pancreas. Furthermore, right pleural dissemination was present.

Macroscopically, two black tumor nodules were observed in the liver (Figure 3H); however, microscopic examination revealed that these consisted of necrotic tumor cells. Additional metastases were found in the bladder and ileum. Although numerous brain calcifications-attributed to radiation treatment for leukemia-were noted, tumor metastasis was not detected in the brain. The cause of death was determined to be hemorrhagic shock due to bleeding from the duodenal metastases.

Disease association regarding next-generation sequencing (NGS) findings was 88% in NF1 [2]. We performed targeted NGS on 275 genes using duodenal tumor samples. The specific cancer-related genes analyzed are listed in a table in the Appendices. Tumor tissues were excised from thinly sliced histopathological sections of each biopsy specimen, and DNA was extracted. The QIAseq Human Comprehensive Cancer Panel (Qiagen, Hilden, Germany), targeting the 275 most commonly associated cancer genes, was used. Mutations in NF1 and KRAS were detected in the sample, both of which are classified as pathogenic in the COSMIC and ClinVar databases (Table 1).

**Table 1:** Gene mutations of NF1 and other pathogenic mutation in duodenal malignant melanoma ^*^Stop codon

Gene	Mutations	Amino acid mutations	Allelic frequency	Clinical significance
NF1	c.380G>A	p.Gly127Glu	0.013	Uncertain significance
NF1	c.702G>A	p.Leu234Leu	0.553	Benign
NF1	c.2034G>A	p.Pro678Pro	0.609	Benign
NF1	c.3094T>C	p.Cys1032Arg	0.187	Likely pathogenic
NF1	c.3100G>T	p.Glu1034^*^	0.352	
NF1	c.4249G>A	p.Ala1417Thr	0.214	Uncertain significance
KRAS	c.436G>C	p.Ala146Pro	0.412	Pathogenic

## Discussion

NF1 is a hereditary disorder that causes lesions in multiple organs. It is inherited in an autosomal dominant manner and occurs in approximately one in 3,000 to 4,000 individuals, with about 42% of mutations arising de novo [3,4]. The NF1 gene, located on chromosome 17q11.2, encodes the tumor suppressor protein neurofibromin, which negatively regulates the Ras/MAPK and PI3K/mTOR signaling pathways [3]. Several thousand pathogenic NF1 variants have been identified in affected individuals [5]. Recent studies have suggested that NF1 mutations also occur in patients with MM. The NF1 tumor suppressor gene plays a role in regulating growth in neural crest-derived cells, including melanocytes, and is mutated in some cutaneous melanomas [6].

MM has been reported in 43.6% of patients with NF1, although the overall risk remains incompletely understood. In patients with conjunctival melanoma, which involves dysregulation of the MAPK and PI3K/AKT/mTOR pathways, mutations in BRAF, NRAS, and NF1 are commonly observed, while KIT and PTEN mutations occur less frequently [7,8]. The Cancer Genome Atlas Network classifies cutaneous melanoma into four molecular subtypes based solely on genetic variation: BRAF-mutant (52%), RAS-mutant (28%), NF1-mutant (14%), and triple wild-type (6%) [9]. Foster et al. [6] reported that 47% of uveal melanomas showed weak neurofibromin expression by immunohistochemistry, and Honavar et al. [10]. described a case of iris melanoma in a patient with neurofibromatosis.

MM primarily spreads through hematogenous metastasis and, similar to neuroblastoma, can metastasize to virtually any organ. Pathologically, hematogenous metastasis is classified into portal vein type, vena cava type, and arterial type, which partly determines the metastatic destination [11]. Autopsy findings indicate that MM often spreads extensively with multiple metastatic foci, ultimately leading to death [11]. MM frequently metastasizes to the gastrointestinal tract, particularly the stomach and duodenum [12-15]. Metastatic MM may be detected early or several years after local excision, with some reports noting liver metastases occurring simultaneously with gastrointestinal metastases [16-18]. In our case, metastases were observed in both the liver and duodenum. It was suggested that the duodenal tumor was a metastasis from the choroidal melanoma, although autopsy findings also raised the possibility that it was a de novo neoplasm. The possibility of a de novo neoplasm was suspected, given the tumor’s spread, increased pleomorphism of the tumor cells, presence of mitotic figures, and obscured melanin deposition. However, because the immunohistochemical characteristics were the same, it was difficult to determine pathologically whether the lesion was metastatic or de novo.

Although it is difficult to differentiate between primary and metastatic MM of the duodenum based solely on medical history [19], distinguishing primary intestinal melanoma from metastatic melanoma is also challenging because cutaneous primary lesions often regress and disappear [20]. Primary melanoma of the small intestine typically presents as a single lesion accompanied by intestinal bleeding and progressive obstruction. By contrast, metastatic cases more commonly involve multiple lesions and often present with acute abdominal symptoms due to intestinal entrapment or obstruction as the initial manifestation [21, 22]. In our case, the duodenal lesion was not localized, as is typical of metastatic lesions involving multiple organs. Instead, it extensively occupied the duodenum and appeared to infiltrate surrounding tissues, including the pancreas. Pathologically, it was difficult to determine whether the lesion was metastatic or de novo. Anvari et al. [23] reported that primary duodenal MM often presents with an ulcerative appearance. However, extensive ulcerative infiltration has also been observed in metastatic duodenal lesions [24], making it difficult to distinguish between de novo and metastatic lesions based solely on macroscopic findings.

Zhou et al [19]. described two patients: one diagnosed with primary duodenal MM and the other with metastatic duodenal MM. Both patients were examined for the BRAF V600E mutation, and the patient with primary duodenal MM tested positive, with a T > A substitution at position 1799 of exon 15. In our patient, six types of NF1 mutations and a pathogenic KRAS mutation were identified, as shown in Table 1, based on the duodenal MM sample. No BRAF mutation was detected. A PubMed search revealed no prior reports of NF1 mutations in duodenal MM. KRAS mutations are present in up to 2% of MM [25] and are integral to the development of melanoma in a mouse model [26].

The main symptoms of NF1 include skin findings such as café-au-lait spots and neurofibromas, with various lesions appearing over time in the nerves, eyes, and bones. Diagnostic criteria include the presence of six or more café-au-lait spots, allowing for a clinical diagnosis in suspected cases without the need for genetic testing. However, genetically, loss of SPRED1, as reported by Brems et al. [27], can also result in tumors that produce an NF1-like phenotype. Therefore, differential diagnosis is necessary to distinguish NF1 from the autosomal dominant NF1-like disorder caused by mutations in the SPRED1 gene, although our panel did not include SPRED1.

In the present case, the patient had café-au-lait spots and was clinically diagnosed with NF1, despite having no family history of the disorder. He also had a childhood history of acute lymphoid leukemia, though further details were unavailable. Because the patient’s leukemia had been treated at another hospital, we do not know the details of his treatment and response to it. However, we do know that his leukaemia had been diagnosed at age one and that he had received treatment until age 19, when he had been in complete remission. A recent study suggested that NF1 may be associated with leukemia, particularly juvenile myelomonocytic leukemia, which has been reported in connection with NF1 [28], indicating the need for more detailed investigations. In the present case, the possibility that an NF1 abnormality caused the leukemia is merely a speculation. Additionally, our patient exhibited borderline intelligence and autism spectrum disorder, which have also been linked to NF1. In NF1, overactivation of RAS signaling can lead to excessive release of γ-aminobutyric acid, disrupting the balance between neuronal excitation and inhibition and potentially contributing to symptoms of autism spectrum disorder [29]. In addition to visible signs such as skin manifestations, NF1 may also be associated with psychiatric disorders and life-threatening tumors.

While NF1 cannot yet be completely cured, advances in medicine have made symptomatic treatment possible, helping to alleviate patient suffering. It is important for patients and their families to understand that all symptoms are the result of genetic abnormalities in NF1. The hallmark features include neurofibromas and an increased risk of malignant peripheral nerve sheath tumors and optic pathway gliomas [30]. The high prevalence of these tumors, along with their potential for malignant transformation and loss of the SUZ12 gene, part of the NF1 microdeletion region, are predisposing factors that increase the risk of malignant peripheral nerve sheath tumors in patients with large NF1 deletions [30]. Patients with NF1, in addition to visible symptoms, must be closely monitored for associated psychiatric conditions and serious tumors. Although a complete cure remains elusive, ongoing medical advances offer hope for symptom management, underscoring the importance of understanding that all manifestations stem from NF1-related genetic mutations.

Our patient had no BRAF mutation, and molecularly targeted therapy with nivolumab and ipilimumab was administered for the treatment of liver metastases. The liver tumor showed necrosis, indicating a therapeutic effect from the targeted therapy. However, the duodenal tumor continued to proliferate despite treatment with pembrolizumab and dacarbazine. MEK inhibitors are expected to be effective for MM with NF1 mutations [31]. Although trametinib is known to be highly effective against BRAF/NRAS wild-type melanoma, its sensitivity in NF1-negative melanoma cell lines is comparable to that in NF1-expressing lines [32], suggesting that further research is required.

## Conclusions

We presented a case that appeared to be linked to a common NF1 mutation. The patient developed multiple illnesses from an early age and struggled with these conditions until he ultimately died of MM at a young age. While the duodenal MM was initially considered a metastasis from the ocular lesion, the possibility that it was a de novo tumor arising from the NF1 mutation could not be excluded. MM has been reported in 43.6% of patients with NF1, although the overall risk remains incompletely understood. Nevertheless, patients with NF1 should be carefully monitored for the potential development of MM. Further research is warranted to clarify the correlation between NF1 mutations and MM.

## Appendices

**Table 2:** Cancer panel target 275 gene list

ABL1	BCL2L1	CDK12	DOT1L	FBXW7	HIST1H3B	KMT2C	MYC	PIK3CA	RHEB	STK11
ACVR1B	BCL6	CDK4	EED	FGF4	HNF1A	KMT2D	MYCL	PIK3R1	RHOA	SUFU
AKT1	BCOR	CDK6	EGFR	FGF6	HOXB13	KRAS	MYCN	PIK3R2	RIT1	SUZ12
AKT2	BCORL1	CDKN2A	EGLN1	FGFR1	HRAS	LRP1B	MYD88	PIM1	RNF43	TAL1
AKT3	BCR	CDKN2B	EP300	FGFR2	HSP90AA1	MAP2K1	NF1	PLCG1	ROS1	TCF3
ALK	BIRC3	CDKN2C	EPAS1	FGFR3	ID3	MAP2K2	NF2NF2	PMS1	RUNX1	TERT
AMER1	BLM	CEBPA	EPHA3	FGFR4	IDH1	MAP2K4	NFE2L2	PMS2	SDHB	TET2
APC	BRAF	CHEK1	EPHA5	FH	IDH2	MAP3K1	NFKBIA	POLD1	SETBP1	TGFBR2
AR	BRCA1	CHEK2	ERBB2	FLCN	IGF1R	MAP3K14	NKX2	POLE	SETD2	TNFAIP3
ARAF	BRCA2	CIC	ERBB3	FLT3	IKZF1	MAPK1	NOTCH1	PPM1D	SF3B1	TNFRSF14
ARID1A	BRIP1	CREBBP	ERBB4	FLT4	IKZF3	MCL1	NOTCH2	PPP2R1A	SMAD2	TP53
ARID1B	BTK	CRLF2	ERG	FOXL2	IL7R	MDM2	NOTCH3	PRDM1	SMAD4	TRAF3
ARID2	CALR	CSF1R	ESR1	FUBP1	INHBA	MDM4	NPM1	PRKAR1A	SMARCA4	TSC1
ASXL1	CARD11	CSF3R	ETV6	GALNT12	IRF4	MED12	NRAS	PRKDC	SMARCB1	TSC2
ATM	CBL	CTCF	EXO1	GATA1	JAK1	MEF2B	NSD1	PRSS1	SMC1A	TSHR
ATR	CBLB	CTNNA1	EZH2	GATA2	JAK2	MEN1	NTRK1	PTCH1	SMC3	U2AF1
ATRX	CBLC	CTNNB1	FAM175A	GATA3	JAK3	MET	NTRK2	PTEN	SMO	U2AF2
AURKA	CCND1	CUX1	FAM46C	GEN1	KAT6A	MITF	NTRK3	PTPN11	SOCS1	VHL
AURKB	CCND3	CXCR4	FANCA	GNA11	KDM5C	MLH1	PAK3	RAC1	SOX2	WHSC1
AURKC	CCNE1	CYLD	FANCC	GNAQ	KDM6A	MPL	PALB2	RAD21	SOX9	WT1
AXIN1	CD274	DAXX	FANCD2	GNAS	KDR	MRE11A	PAX5	RAD50	SPOP	XPO1
AXIN2	CD79A	DDR2	FANCE	GREM1	KEAP1	MSH2	PBRM1	RAD51	SRC	XRCC2
B2M	CD79B	DICER1	FANCF	GRIN2A	KIT	MSH6	PDGFRA	RAF1	SRSF2	XRCC3
BAP1	CDC73	DNM2	FANCG	H3F3A	KMT2A	MTOR	PDGFRB	RB1	STAG2	ZNF217
BCL2	CDH1	DNMT3A	FAS	HGF	KMT2B	MUTYH	PHF6	RET	STAT3	ZRSR2
